# Somali women’s view of physical activity – a focus group study

**DOI:** 10.1186/1472-6874-14-129

**Published:** 2014-10-23

**Authors:** Gerthi Persson, Amina Jama Mahmud, Eva Ekvall Hansson, Eva Lena Strandberg

**Affiliations:** Blekinge Centre of Competence, SE-371 81 Karlskrona, Sweden; Department of Clinical Sciences in Malmö/Family Medicine, Lund University, Jan Waldenströms gata 35, SE-205 02 Malmö, Sweden; Department of Clinical Sciences in Malmö/Social Medicine and Global Health, Lund University, Jan Waldenströms gata 35 SE-205 02, Malmö, Sweden

**Keywords:** Migration, Focus group, Physical activity, Primary health care, Somalia, Women

## Abstract

**Background:**

Physical inactivity presents a major public health challenge and is estimated to cause six to ten percent of the major non-communicable diseases. Studies show that immigrants, especially women, have an increased risk of non-communicable diseases compared to ethnic Swedes. Somali immigrant women have increased rates of overweight and obesity, low fitness levels and low levels of cardiorespiratory fitness compared to non-immigrant women. These findings suggest that Somali women are at increased risk of developing lifestyle-related diseases. Few studies explore determinants of physical activity among Somali women. The aim of this study was to explore Somali women’s views and experiences of physical activity after migration to Sweden.

**Methods:**

A qualitative focused ethnographic approach was used in this study. Four focus groups were conducted with twenty-six Somali women ranging from 17 to 67 years of age. Focus group discussions were recorded, transcribed verbatim and analysed using qualitative content analysis.

**Results:**

The analysis resulted in four main themes and ten categories: **Life in Somalia** and **Life in Sweden, Understanding and enhancing health** and **Facilitators and barriers to physical activity**. Great differences were seen between living in Somalia and in Sweden but also similarities such as finding time to manage housework, the family and the health of the woman. The extended family is non-existent in Sweden, making life more difficult. Health was considered a gift from God but living a healthy life was perceived as the responsibility of the individual. Misconceptions about enhancing health occurred depending on the woman’s previous life experience and traditions. There was an awareness of the importance of physical activity among the participants but lack of knowledge of how to enhance activity on an individual basis. Enhancing factors to an active lifestyle were identified as being a safe and comfortable environment.

**Conclusions:**

Some barriers, such as climate, lack of motivation and time are universal barriers to an active lifestyle, but some factors, such as tradition and religion, are distinctive for Somali women. Since traditional Somali life never involves leisure-time physical activity, one cannot expect to compensate for the low daily activity level with leisure-time activity the Swedish way. Immigrant Somali women are a heterogeneous group with individual needs depending on age, education and background. Tailored interventions with respect to Somali traditions are necessary to achieve an actual increase in physical activity among migrant women of Somalian origin.

## Background

Non-communicable diseases, (NCDs) are responsible for two-thirds of all deaths globally in 2011, and World Health Organization, (WHO) predicts an increase by 15% globally between 2010 and 2020 [1]. Most NCDs are strongly associated and causally linked with four behaviours: tobacco use, physical inactivity, unhealthy diet and the harmful use of alcohol [1]. One third of the adult population is physically inactive causing a risk of overweight and obesity [2]. Obesity is a problem worldwide, spreading in all socioeconomic groups in society. Evidence shows a strong connection between an increase in weight and a low physical activity level in relation to food intake [3]. Several studies indicate that immigrant women show an increased prevalence of unhealthy behaviours and risk of poor health status [1, 2]. The sedentary lifestyle can be a remnant from their country of origin or might be brought about by stressful migration and acculturation into a new social and cultural environment [2]. Somali immigrant women have increased rates of overweight and obesity, low fitness levels and low levels of cardiorespiratory fitness compared to non-immigrant women [4]. These findings suggest that Somali women are at increased risk of developing lifestyle related diseases [5]. It is therefore important for primary healthcare practitioners to facilitate healthy behaviours such as an increased level of physical activity to prevent the risk of developing disease.

Individuals who emigrate from their country of birth are generally healthier than those who do not. However, the healthy immigrant effect tends to wear off with time [6]. For decades Somalia has been affected by catastrophic events due to civil war [7]. Millions of its people have been left displaced and the process of migration, sense of dislocation and alienation contributes to stress. Limited data are available about risk factors of Somali immigrants, but the group experience major changes in lifestyle and face a number of challenges when adapting to their new country, making them more vulnerable to poor health [8]. The Somali population has made a transition, from a primarily pastoral and nomadic to a more sedentary lifestyle with a dramatic change in the daily pattern of physical activity [5].

During the past 10 years 35,100 Somali natives sought asylum in Sweden, a country with 9.5 million people. In 2012 there were 43,966 people from Somalia living in Sweden, 75 percent of them over the age of 16 and under 40 [9, 10].

Some studies offer valuable insight into Somali women’s need for health professionals to accommodate cultural beliefs into health prevention [11–13]. Studies from the Unites States and New Zeeland explore determinants of physical activity among Somali women, and identify barriers and issues concerning designing physical activity programmes [5, 8]. 

However there is a lack of in depth information and understanding of the groups’ preconditions to increase physical activity levels and reduce sedentary behaviour. The aim of this study was therefore to explore and understand the perception of physical activity among Somali women.

## Methods

### Design and sample

To get a holistic understanding of Somali women’s views on physical activity and health, we chose a focused ethnographic approach [14]. The main component of the theoretical position of focused ethnography is the emphasis on understanding the participants’ experiences. Unlike traditional ethnography which requires prolonged periods of data collection in the field, the study focused examining specific issues in a single social situation among a limited number of people within a defined period of time [15]. The principle of holistic understanding implies that people’s views and actions must be understood within the context as well as the time and space in which they occur [16].

Snowball methods were used to recruit participants [16]. Key persons in Somali women associations in the Southern region of Sweden played a key role in identifying and recruiting participants in the different towns. Inclusion criteria were, women over the age of 18, born in Somalia and living in Sweden. Leaflets with information about the study aims and contact information for researchers in Somali and Swedish languages were handed to the key persons in the women’s associations. A date and time for the focus group discussions was set with the help of the same key people. Women who met the criteria and who turned up at the specific time, participated in the focus groups in respective city, thus no specific screening was performed. A total of 26 women between 18 and 67 years participated in four focus groups in three cities; one large city of 338 000 inhabitants and two smaller cities of 60 – 114 000 inhabitants in the south of Sweden. Demographic data are reported in Table 1. Majority of participants knew each other but there were few who had never met before.

**Table 1:** **Participant characteristics**

	Group 1 large city N = 7	Group 2 large city N = 6	Group 3 small city N = 5	Group 4 small city N = 8
Age	42 ± 3.2	26 ± 2.0	31 ± 2.6	39 ± 14.3
Years in Sweden	20 ± 5.8	4 ± 2.3	9 ± 3.4	13 ± 8.3
Somali primary language	7	6	5	8
Single	4	3	2	3
Employed	6	0	0	4
Student	1	6	5	4

Mean ± SD.

Focus group discussion methodology was chosen as it seeks explanations of social and cultural phenomena based on the perspectives and experiences of people being studied. It is therefore compatible with the ethnographical approach adopted in this study [16]. A focus group conversation also invites discussion through participation and often results in groups dynamic which according to Morgan, generates data that is rich in viewpoints as the lively collective interaction can provoke more spontaneous expressive emotional opinions than an individual interview [17].

### Data collection procedure

Data was collected using a semi- structured interview guide under a period of 4 months in 2012 and 2013. Open ended questions were used to allow a fluid conversation about the topic [18]. The interview guide was inspired by Krueger’s practical guide how to conduct focus groups, [19] and Halcomb et al. how to undertake focus group research with culturally and linguistic diverse groups [20]. The interview-questions were developed based on experience and pre-understanding as a physiotherapist that Somali women appear to have a low level of physical activity. The questions were piloted to check for cultural appropriateness and linguistic understanding prior to conducting the focus groups [20]. Venues for the focus groups were chosen by participants themselves. In the largest city, one focus group was conducted at the Somali Community Centre, the remaining two groups were held in the homes of two participants. In the smallest city the focus group was held at the school where the participants study Swedish language.

The participating women had different proficiency on speaking Swedish; a few preferred to speak Swedish and the majority preferred to speak Somali thus the instruction was to discuss the topic in the language of their choice. All focus groups were held by AJM, a nurse and bilingual moderator of Somali descent with a PHD degree and significant knowledge of conducting focus groups. When choosing a facilitator of focus group with a diverse cultural background the person need to be respected, credible and preferably a member of the cultural group [21, 22]. GEP, a physiotherapist and a Swedish native, kept field notes of the interactions occurring and ensured that everyone had the opportunity to speak.

The focus of the discussion was ‘views of health and physical activity’. After an opening presentation, health was introduced in Somali and in Swedish, as the first topic, and the participants were invited to share their thoughts. Health was first discussed since the concept is similar in Somali culture. The concept of physical activity needed some explanation and was defined as any bodily movement associated with work, during transportation or leisure time. Following the initial questions a discussion took place with focus on the relation between health and physical activity, experience of physical activity, enablers as well as barriers to be physically active. Based on the discussions, we tried to elicit shared thoughts, opinions, and a meaning that could increase our understanding of how Somali women viewed health and physical activity. Once each topic was discussed AJM made a summary in Somali to the respondents to verify the statements. A post meeting of the session was held by AJM and GEP after every focus group to discuss the outcome of the discussions and to identify new ideas that need to be exploited in the next focus group [19]. A brief summary in Swedish was also made at the end of the session by AJM.

The focus group discussions lasting between 75–90 minutes were recorded digitally and everything said in Swedish was transcribed verbatim by a native Swedish secretary. The Somali parts of the interviews were transcribed verbatim by a secretary. The secretary, who is a native Somali with nursing background and a broad experience in transcribing and translating interviews, translated the data into English. The translation was validated by AJM and later discussed with GEP in line with suggested steps of translation process to ensure quality [23]. Field notes of observations were completed by GEP following the focus groups to complement interview transcriptions in Swedish and English. The use of field notes provided additional information about the non-verbal exchange of information between the participants.

### Analysis

Qualitative content analysis was used to analyse the material [24]. To familiarize with the data, GEP and AJM read and discussed the summaries, translations and field notes and when supplementary verification was needed, listened to the recordings. ELS, a behavioural scientist thoroughly familiar with focus groups, read through the Swedish and English transcriptions several times separately. Combining the interviews and observational notes provided depth and allowed the researchers to understand the experience and processes. The analysis followed an inductive approach and meaning units were identified by two researchers as a first step. Line by line coding was completed across all data collected and expressed by the participants.

The codes were grouped into general categories with shared content [24]. Categories and themes were developed on the basis of the codes. Several strategies were applied to ensure trustworthiness of the findings [24]. The text was analysed individually by the authors. Two of the authors (GEP and AJM), met several times and participated in all steps. ELS participated in developing sub-categories, categories and themes. EEH, a physiotherapist, read all the material, reflected, commented and confirmed that they contained data supporting the findings. Triangulation was accomplished through multiple methods of data collection including field notes and interviews. Examples of the analysis are seen in Tables 2 and 3.

**Table 2:** **Life themes with meaning units, codes and categories derived from the analysis**

Meaning units	Code	Category	Theme
“Women cooked together, they spent time together and got along. Life was simple.” P13*	**Household chores were done together**	**Extended family essential for everyday life**	**Life in Somalia**
“Migration has changed everything, stress, children and large family to care for.” L15*	**Alone with a large family**		
“When you come to a new country and you don’t have support or someone helping you, it’s easy to be affected by stress.” K7*	**Stressful life without support**		
“If you are sick here in Sweden, you still have to do everything yourself, whereas in Somalia you get help.” P15.	**No help when needed**		
“My situation is different because I wasn’t married off, I chose the person I wanted to get married to. So I didn’t have the support from my family.” P16	**Go against the will of the family**	**Traditional way of living**	
“The house work takes most of the time and energy. You have to go to the store and buy fresh things and then go home and cook the same thing every day, walk back and forth, buy the things and come back.” P12	**Household chores occupy the whole day**		
“I’m tired and afraid. Life in Sweden is stressful! There is always something emerging.” P18–19	**Always having something to do is stressful**	**Life filled with chores and no spare time**	**Life in Sweden**
“And all this stress, the sugar levels start because we have not adapted to this country. As a result you get health issues.” K3	**Hard to adapt to a new life**		
“The house work takes most of the time and energy.” P12	**Household chores takes all energy**		
“It is difficult for a single mother and if maybe the kids’ father lives with her she might get some help”. L15	**Maybe help if the spouses are living together**		
“I went to school this morning, and when I finish I will pick up the children from school. When my husband comes home I will leave again for an appointment at the school with the teacher, even though I have other things needing to be done.” K3	**A structured life with many appointments**		
“The little time we have to ourselves we go for tea to somebody’s house and we take tea with a lot of sugar.” K7	**Spare time is spent drinking tea**	**Traditional culture prevails**	
“We cannot leave our cultural background with regard to food.” L7	**Traditional food is consumed**		
“The household chores and the kids, where would I get time for physical activity? The walks and movements that I do are enough.” K8	**Physical activity doing household chores**	**No further need to move**	
“As a mother with full-time activity there is no spare time, my schedule is packed. I am very tired cooking, washing etc. I do not need any physical activity.” K7	**Full-time activity taking care of the house**		

*K: focus group 3, small city; L: focus group 4, small city; P: focus group 1 and 2, large city.

**Table 3:** **Health and Physical activity themes with meaning units, codes and categories derived from the analysis**

Meaning units	Code	Category	Theme
“We believe in God, he is the one who grants health.” K4*	**Health is a gift from God**	**Faith in God**	**Health**
“We have thick skin; the sun can penetrate through our skin.” L12*	**The sun can penetrate our skin**	**Misconceptions**	
“My mother used to drink juice with plenty of sugar and tea with a lot of sugar. In Somalia you burnt the calories so my mother had good health.” L14	**Good health despite high intake of sugar**		
“If I take a walk outside or go to some activity and jump and sweat, my blood circulation and everything works and health also comes.” L4	**Jump and sweat is healthy**	**Enhancing health**	
“If I do not work out for a long time I cannot even sleep.” L8	**Bad sleep due to no movement**		
“If gyms are mixed men and women we cannot go, but if the gym is for women only, we could go.” L8	**Gym for women only is a must**	**Facilitators**	**Physical activity**
“If somebody ‘holds your hand’ supports you and motivates you, you walk!” K16	**Support is motivational for walking**		
“I love swimming but my religion does not allow men to mix with men.” K12	**Religion does not allow mixing with men**	**Factors affecting physical activity**	
“It is winter and it’s too cold to walk, and it’s dark.” P10*	**Restrained from walking due to a cold and dark environment**		
“To get dressed and walk alone I see as the most boring thing ever!” K15	**Walking is boring**		
“I have the chance to work out, but we Somalis would choose to go to a friend’s house and drink tea with a lot of sugar and chat. There are health benefits in chatting too.” L16	**Chatting takes priority over a workout**		

*K: focus group 3, small city; L: focus group 4, small city; P: focus group 1 and 2, large city.

### Ethical considerations

Ethical approval was granted by the regional ethics board in Lund, registration number 2011/517. The aim of the study and the focus group methodology were presented in Somali and in Swedish in the information letter and informed consent was obtained from the participants. The Somali women took part voluntarily in the study and were informed of their right to end their participation at any time. The material was de-identified and coded to guarantee confidentiality.

## Results

### Summary of results

Twenty-six women born in Somalia living in Sweden participated in four focus groups. The average time living in Sweden was 11.56 years, ranging from 1 to 23 years (Table 1). There was a wide age range of women in the focus groups but their views on health and physical activity did not differ. The overall analysis shows that the participants were aware that physical activity ought to be an important component of a healthy life but pointed out different obstacles. The results also showed that there were possibilities to become physically active if support and enthusiastic leaders were available. Four main themes and ten categories emerged from the analysed material: **Life in Somalia** and **Life in Sweden**, **Understanding and enhancing health** and **Facilitators and barriers to physical activity** (Tables 2 and 3).

### Life in Somalia and life in Sweden

Migration to Sweden involves a number of stressors and strains that affect adaptation to the new environment. Living in a hot climate forces people to move with less intensity and the traditional lifestyle is considered to be sedentary. Living conditions in Somalia are totally different from life in Sweden. In both countries similar daily routines such as grocery shopping, cooking and cleaning exist, but the participants’ views of what it is like to live in the two countries were not coherent. In Somalia chores are done together with other women and an extended family is always there to help, whereas in Sweden every woman alone takes care of her own household. Even though living in Sweden means having access to household machines such as vacuum cleaners and dishwashers, some participants thought that daily housework takes more time and energy when living in Sweden.

Living together with an extended family, sharing the daily housework with other women, was viewed by some participants as being simple and easy. Other participants felt that living as close together as one does in Somalia can sometimes put a strain on relationships. Life in Somalia can be easy as long as you get along with people you live with; otherwise it brings a lot of stress and difficulties. Being new to a country, without the support of your family, makes life stressful. The women expressed a loss of the extended family when moving to Sweden:

“You have family there [in Somalia] that can help you. But here [in Sweden] there is nobody helping you because the family is not here”.

Life in Sweden is considered very stressful and filled with tasks to do from early morning to late at night, or as one participant put it:

“Lifestyle here is like herding goats: taking kids to school, going to work, bringing them back, and preparing a meal. Where does a mother get time to go to the gym?”

The women stated that taking care of the household without an extended family takes more time and energy than before. Some participants found life in Sweden difficult since they never had to do dishes or cook a meal before moving from Somalia. Household chores were done by a maid, someone else in the household, or the participants were just too young to be responsible for the daily activities before migrating.

Shopping for groceries and cooking is done on a daily basis in Somalia. People walk long distances in order to buy household goods. In Sweden supermarkets and refrigerators make it possible to do shopping once a week, a lifestyle adopted by the participants. This new lifestyle robbed the participants of valuable daily physical exercise, according to participants.

Food and beverages were identified as a potential barrier to a healthy lifestyle. Traditional Somali food contains a lot of sugar and the participants know it is bad for health. Participants continue with their traditional consumption of the “typical” Somali staple food and beverages such as sweet tea, cookies, rice and meat, while the consumption of vegetables and other greens has been reduced. According to the participants, grocery shopping in Sweden is difficult since it is hard to recognize the packaging of the product needed for cooking. In big supermarkets fruit and vegetables lack the typical scent, and spinach, for instance, looks different from the spinach found in Somalia. They are also aware that the level of physical activity and the intake of food must be changed, but traditions are a part of one’s identity and become more important when moving to a foreign country.

### Understanding and enhancing health

Health means different things to different people, depending on the situation. In general it is the condition of a person’s mind and body, usually meaning being free from illness. Health was described as being able to do everything a person desires to do and it was considered a gift from God to achieve and to nurture. Each person is responsible for his or her own health, and everyone could make choices in life to enhance or to spoil health, causing mental and physical illness. Socialization among friends gives peace of mind and may not increase physical health but contributes to enhancing mental health:

“I think the mind needs conversation, a cup of tea, dates and Somali sweets.”

Several times during the discussions, explanations were sought to understand symptoms and conditions where prior health care visits had failed to give clarifying answers, and this led to unfounded anxiety. Somali traditions and views of health differ from the way Swedes perceive factors affecting health, and the understanding of health is based on experience, tradition and misconceptions. A question came up why high blood pressure in Somalia was present even though the country has an abundance of sunlight, and one participant argued for her belief:

“The sauna is a hot place and if you sit there for a long time you sweat a lot, and sugar and fat and toxins come out of your body.”

Several women argued that reliance on health professionals is the greatest when medication is given:

“The Somalis love tablets, even if the doctor says the best medicine for blood pressure is walking, and making the body sweat, and eating a lot of vegetables, we just love medication, it does not matter what part of the body is aching”.

### Facilitators and barriers to physical activity

Physical activity was considered important to health and well-being, and the discussion identified both facilitators and barriers to being physically active. In Somalia physical activity is incorporated into activities of daily living. Walking for health or just for the sake of it never existed. One participant talked about how Somalis think about walking:

“Unless we force ourselves it is not in our mind to walk.”

Adults never engage in leisure-time activity, it is for children only. Leisure-time activity for a Swede often involves physical activity, something totally unfamiliar to a person from Somalia who uses spare time to sit at home, or as one participant explains:

“We make very sweet cakes or cookies, sit facing each other and that is what the majority of us do.”

Participation in leisure-time physical activity had occurred since they moved to Sweden. The activity became a positive experience when it was organized in such a way that the requirements of Somali customs were met and when a fellow Somali woman became an informal leader. One of the participants expressed a sense of well-being following the activity along with nights of sleeping well. The successful activity was run by an enthusiastic Somali woman who mobilized a whole group for jogging activity on a weekly basis, and with her energy was she able to mobilize a whole group. Dressed in traditional clothing, the group had a fixed day to meet at a nearby schoolyard. The importance of creating a comfortable and safe environment was emphasized in order for the participants to move freely. Physical activity is preferably performed separately from men, but a minority could consider going to a gym with men and women mixed. Being physically active is for many women equivalent to spending time at a gym. The possibility for other people to watch while they are exercising can create an uncomfortable feeling and exercise is therefore avoided. Depending on where you live you might have access to a gym for women only, giving the opportunity to join the health club and work out a couple of hours once a week together with friends and other women.

Choosing a familiar place for the exercise is important, and if a public place is chosen it should be well lit and equipped with cameras for surveillance in order to create a sense of security. The companionship of other women was a major facilitator to maintaining the habit of being physically active. The need for support from someone who can motivate and keep up the spirit of exercising was identified, or as one woman described her situation:

“I would personally need somebody helping with the chores or go to a health camp Somali Biggest Losers.” [Like the Swedish television show, Biggest Loser]

One important view expressed by many women was that life in Sweden gives no incentive to go outdoors or even to get dressed sometimes, unless there are children in the household attending school, or if you have work to go to or as one woman explained:

“Nowadays, I usually think why should I get dressed and go outside if I do not need to go and buy milk like I did in Somalia every day.”

When you need to go outside, the choice of walking or using public transportation is easy, since the bus is always available, taking you wherever you need to go.

Single mothers, as well as participants living in a relationship, mentioned a busy schedule, with no time or energy to be physically active more than everyday chores require. The cold climate in winter is a major barrier to going outside, and thus leaving the home is easier in the summertime. The custom in Somalia is to stay indoors and sleep on rainy days. Along with the cold climate, the darkness is a hindrance to walking outside. Fear of walking alone, in the dark, as well as feeling unsafe in the neighbourhood are reasons for spending a lot of time indoors.

Religious and cultural factors also affect a physically active lifestyle. Traditional Somali clothing for women, a full-length dress and a hijab, is less suitable when you need to move freely. According to Somali customs, wearing swimwear and mainstream European clothing when males are present is forbidden, “haram”. Besides taking too much effort to get dressed, walking is the most boring activity you can do, especially when you are alone. It is also considered “haram” by some women to move to music.

## Discussion

### Summary of results

Life in Sweden was regarded by Somali women as a mixture of traditions and adapting to a new environment and situation. The participants considered health a gift from God and found it to be the individual’s responsibility to make health-enhancing choices. Physical inactivity is increasing among the world’s population and Somali women are no exception. Factors participants identified as enhancing an increased physical activity were safe surroundings, support from people and the company of fellow Somali women. The importance of having enthusiastic leaders and role models to set a good example for a healthy life was also stressed. Barriers such as motivation, lack of time and climate are universal to mankind. Other identified barriers exist for not being physically active, of which tradition and religion are factors distinctive for the group.

### Discussion of method

The aim of the study was to explore and understand the perception of physical activity among Somali women. We conducted four focus groups to collect data, a number recommended in published studies [25, 26]. Language and cultural barriers must be acknowledged as limitations in this study. The fact that the study is bilingual and represents a cultural diversity is a source to misinterpretation of data [23]. The topic and the main themes were presented to the participants in both Somali and Swedish. The participants could choose to give their views in the language they preferred, resulting in a mixed discussion and a limitation to the non-Somali-speaking researcher co-conducting the focus groups. Another limitation is the procedure of translation [23]. One translator was used instead of a team of bilingual translator thus to compensate the limit we used comparison of translations by researcher and translator cognizant of the customs, values, beliefs and language of the respondents [23]. The moderator and translator were nurses that may have class and educational biases of their shared ethnic culture and social positions that they perceive as superior to the participants. On the other hand clinicians can increase the likelihood of gathering and translating accurate data [27].

The selection was confined to southern Sweden and the participants were invited by snowball recruitment through a contact person in the local Somali community; no specific selection was made. Other Somali women might have different views from the women taking part in this study.

### Discussion of the results

Somali women experienced major changes in lifestyle since immigration and we found that life in Sweden gives no incentive to be physically active and the level of activity in everyday life has decreased.

Findings show that most people tend to stick to their cultural habits and values even more firmly, to retain their original identity in a strange and new environment [28]. The identity issue faced by Somali immigrants depends on age, gender and how much time the person has spent in the host country. The elderly Somalis prefer to remain within their community whereas the younger generation juggles with multiple identities [10]. Social norms, attitudes, customs and gender roles derive from Islamic tradition and religion becomes a way to preserve identity and a sense of community and may work as a moral and practical guide to everyday life [29–31]. Studies have shown that especially women become more religious away from Somalia [10, 29]. Besides religion, the clan system still prevails and influences life by being a source of comfort and at the same time exerting social control [10]. Together with the ability to speak Swedish, it was noticeable that the younger women appeared more outgoing, interested in knowing more about health, physical activity and Swedish manners, yet keeping to Somali traditions and norms always was important. The participants talked about the absence of the extended family to give support and help in everyday life. With this knowledge in mind it is understandable that life in Sweden appears different and stressful.

To treat illnesses, Somalis may use extensive herbal medicine traditions and commonly expect to receive medication for every illness when utilizing Swedish health care systems [31]. Physical activity on prescription (PAP) is used in Swedish health care to promote physical activity, and Swedes show 65 percent compliance with the method although GPs regard PAP with mistrust and use the method sparsely[32, 33]. Women in the study pointed to a great trust in medication, indicating that adherence to a non-pharmaceutical method might be low. Ethnographic guidelines suggest that Somali patients may be disappointed when no pharmaceuticals are prescribed [31].

The conception of health and the factors that facilitate good health are different in Sweden and in Somalia. Depending on whether a person comes from a rural or urban area in Somalia, views of health and how to use medical systems vary [31]. Similar findings were seen among the participants together with a gap of knowledge due to varying levels of education and prior experience.

Regardless of origin, daily physical activity is genetically and biologically regulated and humans always avoid moving more than necessary in order to preserve energy for times when it is better needed [34, 35]. The women showed different levels of motivation for physical activity, just like any person in the process of a change to an increased level of activity. Behavioural changes to a higher level of physical activity are challenging both to the person needing a change and to the supporting health professionals [36, 37]. Participants pointed to enablers crucial for lifestyle changes, such as creating a feeling of comfort, support and companionship with other women, factors universal to everybody trying to increase the level of physical activity. Other populations’ points to similar factors [5, 8] but Somali women appear to have special needs to feel secure and comfortable when being physically active, which requires the activity to coincide with Somali traditions and lifestyle. The great need for comfort might be an indication of a lack of belonging to society. According to unpublished data, the sense of belonging derives from the individual’s willingness to be a part of society but it is also society’s acceptance of the individual that matters [28].

Barriers to being active were elucidated, identical to those seen in previous studies, including climate, religious, cultural and safety concerns [5, 38]. Furthermore the participants talked about traditional Somali food being a risk factor for health, and difficulties in changing diet from traditional Somali food to a healthier alternative are not considered [39].

Life in Somalia and Sweden is as different as can be expected, although similarities exist. Whether women are living in Sweden or Somalia, finding time for the home, family and for the woman herself will always be an issue. Awareness of the importance of physical activity exists, but tools to enhance activity on an individual basis are missing. Identified barriers, such as motivation and lack of time to become more physically active, are universal for every human being. Barriers such as language, religion, values and tradition need to be taken into consideration when planning interventions for Somali women. The decrease of physical activity in everyday life is a global issue, and international recommendations for physical activity address the link between the frequency, duration, intensity, type and total amount of physical activity needed for the prevention of NCDs [2]. It is necessary to focus on changing the sedentary life trend for Somali women as well.

Major life events have a strong effect on leisure PA behaviour [32]. Consequently, Somali women who have experienced substantial life events by migrating are an important target group for physical activity promotion. This study sheds light on Somali women’s views and experiences of physical activity, and there is a need for more information concerning the actual level of physical activity in order to offer adequate support to the group. Healthcare providers must be aware of individual variations but also be wary of generalization that may lead to stereotyping [40]. Other studies have shown, just like this study, that regardless of country of origin that support on different levels is crucial to establish an increased level of physical activity in both short and long term [5, 41, 42]. This study found that Somali women settled in Sweden are not a homogeneous group needing exactly the same type of intervention to adopt a less sedentary lifestyle. Interventions focusing on enhancing physical activity in everyday life could therefore be successful.

## Conclusions

Immigrant Somali women constitute a heterogeneous group with individual needs depending on age, education and background. Several reasons emerged for adopting a sedentary behaviour and facilitators as well as barriers for Somali women living in Sweden were identified to increase and stimulate physical activity. This study is consistent with what is known about general barriers to becoming physically active, but demonstrates the group’s specific needs when addressing interventions to reduce a sedentary lifestyle. The daily physical activity performed in the home country has been lost and replaced with chores filled with convenience, leaving no incentive for daily physical activity. Interventions to increase daily physical activity levels and minimize a sedentary time are important. Primarily, there is a need to raise the awareness of the harmful effect that a sedentary lifestyle pose on health. If the need for a change is recognized the individual is more likely take part in suggested interventions and become ready to change behavior.

Since traditional Somali life never involves leisure-time physical activity, one cannot expect these women to compensate for the low daily activity level with leisure-time activity in the Swedish way. Health programs to facilitate physical activity must consider a safe supportive environment where Somali women can feel secure. The women’s views and experiences will provide ideas for culturally relevant interventions to stimulate physical activity on a daily basis to avoid NCDs. Tailored interventions with respect to Somali traditions are necessary to achieve an actual increase of physical activity among women who have emigrated from Somalia.

## Abbreviations

NCD: Non-communicable disease
PAP: Physical activity on prescription
WHO: World Health Organization.
